# A new era of precision diagnosis and treatment for lung cancer: artificial intelligence-driven multimodal data integration and clinical applications

**DOI:** 10.1038/s41419-026-08769-z

**Published:** 2026-04-22

**Authors:** Na Liu, Guohu Han, Qianhui Gu, Yan Zhang, Minbin Chen

**Affiliations:** 1 Department of Radiotherapy and Oncology, Affiliated Kunshan Hospital of Jiangsu University, Kunshan, China; 2 Department of Oncology, Jingjiang People’s Hospital Affiliated with Yangzhou University, Jingjiang, China

**Keywords:** Cancer screening, Gene therapy

## Abstract

Certain characteristics, such as high heterogeneity, a complex tumor microenvironment, metastatic potential, and drug resistance, render Lung cancer (LC) a formidable challenge for clinical management. With rapid advancements in high-throughput sequencing, medical imaging, and digital pathology technologies, significant amounts of high-dimensional and heterogeneous multimodal data are now being generated. Traditional methods cannot comprehensively elucidate the intrinsic patterns within these multi-omics data, thereby limiting a comprehensive understanding of the biological characteristics of LC. Artificial intelligence (AI) technologies provide powerful computational frameworks for integrating such multi-scale and heterogeneous data. Through cross-modal data integration, AI enables the construction of a panoramic disease atlas ranging from microscopic molecular variations to macroscopic imaging phenotypes. This narrative review aims to systematically discuss the prospects of AI-driven multimodal data fusion in LC research and clinical applications. Our review highlights the potential clinical applications of integrating AI with multi-omics technologies in areas such as early screening, prognostic risk assessment, precision treatment, drug sensitivity analysis, and guiding personalized surgical plans. Against the backdrop of continuous advancements in AI research, we further discuss the main obstacles in translating AI-based multi-omics from research to clinical practice and propose strategic and actionable approaches to promote rapid development in this field.

## Facts

- Clinical Evidence Gap: Can AI tools demonstrate tangible improvements in patient survival and diagnostic efficiency in prospective randomized controlled trials (RCTs), moving beyond high accuracy in retrospective “in silico” validations?
- The “Black Box” Dilemma: How can the field transition from uninterpretable algorithms to Explainable AI (XAI) that visualizes biological rationale (e.g., metabolic nodes, spatial interactions) to secure clinician trust?
- Rise of Foundation Models: Will the paradigm shift from task-specific models to general-purpose Foundation Models (trained on massive unlabeled data) successfully address the scarcity of labeled data in clinical practice?
- Digital Health Integration: The potential of fusing “passive sensing” from wearable devices with “microscopic liquid biopsy” to transform LC care from intermittent hospital visits to proactive, real-time home monitoring.

## Introduction

LC remains the leading cause of cancer-related incidence and mortality in China, characterized by a poor 5-year survival rate of only 19.7% [1–3]. Clinical management is currently constrained by several key challenges, including difficulties in early detection, high tumor heterogeneity, the prevalence of acquired treatment resistance, and high rates of metastasis and recurrence [4, 5]. These persistent clinical hurdles highlight a critical imperative to seek transformative breakthroughs through basic research and translational medicine.

The multifaceted complexity of LC arises not merely from the genomic and epigenetic heterogeneity inherent to tumor cells, but also from the continuous dynamic evolution of the tumor microenvironment (TME) [6–9]. Given the intricate interplay among immune cells, stromal components, and the vascular system, single-omics data are increasingly insufficient to fully recapitulate the comprehensive tumor landscape [6–10]. The integrative analysis of multi-modal data offers a systematic dissection framework in that genomics can delineate driver mutation trajectories; single-cell and spatial transcriptomics can resolve cellular states and regional architectures; pathomics and radiomics can quantify phenotypic characteristics; while metabolomics can facilitate the monitoring of dynamic metabolic flux [8–13]. Spanning scales from micro-molecular to macro-phenotypic, these technologies collectively delineate the underlying mechanisms governing LC tumorigenesis, progression, and immune evasion [14–16]. Nevertheless, the massive, heterogeneous, and highly non-linear nature of data generated by multi-omics integration poses significant challenges for traditional analytical methods in effectively mining intrinsic biological patterns [17–19]. AI leverages robust feature extraction and pattern recognition capabilities to effectively process such complex datasets and systematically decode interaction networks within the TME [17, 20, 21]. Capitalizing on breakthroughs in cross-modal representation learning, self-supervised pre-training, and few-shot generalization, these models efficiently integrate multidimensional information encompassing radiology, proteomics, genomics, and clinical parameters [22–25]. This not only facilitates the excavation of deep biological associations but also supports earlier lesion detection and more precise prediction of therapeutic responses, thereby providing a solid foundation for the advancement of intelligent LC management systems [22–25].

In this narrative review, we systematically elucidate the prospects of AI-driven multimodal data fusion in LC research and clinical applications. We highlight the transformative potential of combining AI with multi-omics technologies in areas such as early screening, prognostic risk assessment, precision treatment, and drug sensitivity analysis. AI-driven multimodal fusion is undoubtedly propelling a profound transformation in the diagnosis and treatment of LC from empirical medicine to data-driven precision medicine, paving new paths for achieving intelligent management across the full life cycle of LC and realizing therapeutic breakthroughs.

## Artificial intelligence and multimodal data

### Evolution of the artificial intelligence

**Fig. 1:**
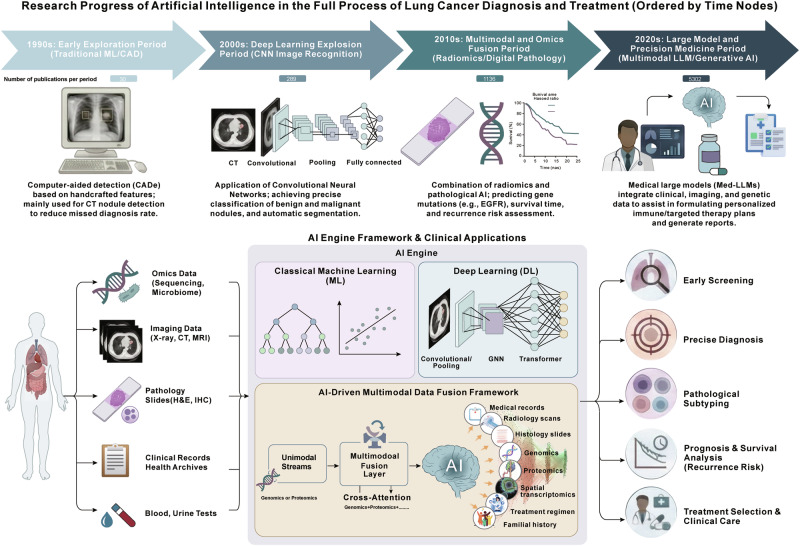
The evolution and application framework of artificial intelligence in the full-spectrum management of LC. The figure outlines the transition from traditional machine learning to multimodal generative AI over four decades (top). The schematic diagram (bottom) demonstrates how the AI engine processes multimodal inputs such as multi-omics data, radiological images, and pathological slides to empower precision oncology across diagnosis, risk assessment, and therapeutic decision-making.

The rapid advancement of AI technologies has provided robust support for addressing complex tasks [26, 27]. Models represented by convolutional neural networks (CNNs) have demonstrated exceptional performance in domains such as image recognition and object detection by enabling the automatic extraction of hierarchical spatial features [26, 27]. In addition, the Transformer architecture and its derivatives (e.g., BERT, ViT) have achieved significant breakthroughs in natural language processing and sequence modeling, effectively capturing long-range dependencies by self-attention mechanisms [28–32]. Furthermore, graph neural networks (GNNs) have exhibited unique advantages for the analysis of protein-protein interaction networks and metabolic pathways [33–35]. These core models not only deliver superior performance but also possess strong scalability and generalization capabilities, thus providing a solid technical foundation for achieving intelligence ranging from perception to decision-making. The evolution and application framework of artificial intelligence in the full-process management of LC are shown in Fig. 1.

### Multimodal data fusion

With rapid advancements in high-throughput sequencing, mass spectrometry, and advanced medical imaging technologies, oncology research has formally entered the era of big data, achieving cross-scale coverage ranging from microscopic molecular mechanisms to macroscopic tissue phenotypes [36–38]. AI models provide unprecedented tools for the integration of multi-modal data derived from tumors [39–41]. By fusing multi-level information encompassing genomics, radiomics, transcriptomics, metabolomics, and proteomics, these models enable the construction of a panoramic tumor landscape spanning from molecules to phenotypes [39–41]. Algorithms such as deep learning are not only capable of individually mining latent complex patterns within each data modality, such as gene mutation signatures, imaging texture regularities, and protein expression networks, but also facilitate the elucidation of non-linear, high-dimensional interaction mechanisms between different levels of biological data through cross-modal associative learning [42–45]. This integrative analysis transcends the limitations of single-omics approaches, facilitating more precise tumor subtyping, drug response prediction, prognostic assessment, and the formulation of personalized treatment strategies, thereby driving a profound transformation in oncology research and clinical practice toward a systematic and precision-oriented paradigm [17, 46]. The key artificial intelligence concepts and architectures relevant to LC diagnosis and research are listed in Table 1.

**Table 1:** Key AI concepts and architectures relevant to LC diagnostics and research.

Omics	AI Category	Concept/Model	External validation	Clinical implementation	Potential clinical applications	References
Radiomics	Machine learning	LASSO, eXtreme Gradient Boosted Trees, and Random Forest	No	No	Predicting pulmonary nodule malignancy	[66]
	Deep learning	Convolutional Neural Network and Vision Transformer -Base (ViT-B)	Yes	No	Term prediction of LC Risk	[69]
	Machine learning	Multivariable Logistic Regression	Yes	No	Identifying malignancy in pulmonary	[71]
	Deep learning	Convolutional Neural Network	Yes	No	Inform LC screening intervals	[70]
	Deep learning	Ensemble of 2D and 3D Convolutional Neural Networks	Yes	No	Assessment of malignancy risk in pulmonary nodules	[68]
Genomics	Machine learning	Google DeepMind, Random Forest, Transformer	No	No	Mutation predictions	[325]
	Deep learning	Convolutional Neural Network	No	No	Mutation predictions	[83]
	Machine learning	Support Vector Machine	No	No	Early diagnosis	[84]
	Machine learning	Logistic Regression	Yes	No	LC detection	[86]
	Ensemble Learning	Random Fores	Yes	No	Detecting pulmonary malignancy	[85]
	Machine learning	Random Forest	No	No	Identify SCLC subtypes	[87]
Proteomics	Machine learning	Random Forest and Support Vector Machine	Yes	No	Early prediction of lung cancer	[88]
Proteomics	Machine learning	LASSO + LightGBM (Light Gradient Boosting Machine)	No	No	Early detection and risk stratification	[89]
	Machine learning	Random Forest	No	No	Profiling Predicts LC	[93]
	Machine learning	LASSO Regression	Yes	No	Diagnosis	[94]
	Machine learning	Support Vector Machine, SVM	Yes	No	Diagnosis	[90]
Pathomics	Deep learning	Deep convolutional neural network	Yes	No	Classification and mutation prediction from NSCLC	[101]
	Deep learning	Deep Multiple Instance Learning (MIL)	Yes	No	Gene mutation prediction	[102]
	Deep learning	Self-supervised Learning	Yes	No	Subtyping and risk stratification	[103]
	Deep learning	Convolutional Neural Network (CNN)	Yes	No	Immune Checkpoint Inhibitor Response Prediction	[105]
	Deep learning	Graph Neural Network (GNN)	No	No	Guiding personalized treatment decisions.	[104]
Single-cell RNA sequencing	Deep learning	Autoencoder, AE	No	No	Predicting the response to immunotherapy	[111]
	Deep learning	Transformer MinCutPool and Graph Attention Networks (GAT)	Yes	No	Guiding personalized treatment decisions.	[112]
	Traditional machine learning	Least Absolute Shrinkage and Selection Operator	No	No	Predicting the prognosis of patients	[113]
	Ensemble machine learning	LASSO and Regression Random Forest	Yes	No	Predicting prognosis and immunotherapy response	[114]
Single-cell RNA sequencing	Machine learning	LASSO	Yes	No	Prognostic stratification	[115]
	Unsupervised machine Learning	WGCNA and LASSO	No	No	Predicting prognosis and immunotherapy response	[116]
	Ensemble machine learning	LASSO, SVM-RFE, and Random Forest algorithms	No	No	Predicting Prognosis and Immunotherapy Response	[117]
	Multi-modal ensemble machine learning	LASSO and Cox	Yes	No	Predicting the risk of lymph node metastasis and response to immunotherapy (TCIA)	[118]
	Ensemble machine learning	LASSO and Random Forest	Yes	No	Prognosis evaluation and personalized treatment guidance	[119]
	Standard statistical machine learning combination	Consensus Clustering and LASSO	No	No	Prognosis evaluation	[120]
	Comprehensive machine learning benchmarking	DeepSurv, RSF, and LASSO	Yes	No	Predicting outcome and immunotherapy response	[121]
Spatial transcriptomics	Machine learning and supervised learning	LASSO Cox	No	No	Prognosis evaluation	[124]
	Deep learning	Deep CNN	No	No	Prognosis evaluation and personalized treatment guidance	[125]
	Machine learning	Bayesian Spatial Deconvolution	No	No	Guiding personalized treatment decisions.	[126]
Metabolomic	Ensemble machine learning	Random Forest and Support Vector Machine	Yes	No	Survival prediction	[129]
Metabolomic	Unsupervised machine learning algorithms	Bisecting k-means clustering	No	No	Subtyping	[132]
	Machine learning	Random Forest, Logistic Regression	No	No	Predicting efficacy of chemoimmunotherapy	[130]
	Deep learning	Attention-based 1D-CNN	Yes	No	Diagnosis	[96]
	Machine learning	SVM and Logistic Regression	Yes	No	Diagnosis	[97]
	Deep learning	Graph Convolutional Layer	No	No	Diagnosis	[98]

To effectively integrate the multi-scale and heterogeneous data streams generated in LC research, AI models utilize distinct architectural taxonomies [47–52].These strategies are fundamentally categorized based on the stage at which information from different modalities is merged. (i) Early Fusion (Data-level Fusion): This strategy involves concatenating raw data or initial feature sets from various sources (e.g., combining clinical parameters with genomic mutation profiles) before feeding them into a single machine learning model [47]. While simple, it often struggles with high-dimensional “curse of dimensionality” and the alignment of modalities with vastly different scales [47]. (ii) Intermediate Fusion (Feature-level Fusion): Currently the most dynamic approach, intermediate fusion transforms each modality (radiology, pathology, omics) into high-dimensional latent representations using modality-specific encoders (e.g., CNNs for images, GNNs for protein networks) [47, 49, 53]. These features are then merged within the neural network layers, allowing the model to capture non-linear, cross-modal correlations [47]. (iii) Late Fusion (Decision-level Fusion): Each modality is processed by an independent model to produce a local prediction or score [47]. These individual outputs are then aggregated—using techniques like “stacking” or weighted averaging—to reach a final clinical consensus [47]. This approach is robust to missing data in one or more modalities but may overlook deep inter-modal interactions [47]. (iv) Attention-based Fusion: Representing the current state-of-the-art, this method utilizes attention mechanisms (e.g., Transformers) to dynamically weight the importance of specific features across modalities [52, 54–57]. For instance, the model may focus more on “pathomics” features when “radiomics” signals are indeterminate, effectively opening a window into the “black box” by visualizing which modality most influenced the decision [52, 54–57].

## Application of artificial intelligence in early screening and diagnosis of LC

In this section, we review the applications of AI-driven omics technologies specifically within the early detection and diagnostic phases of LC. Our analysis encompasses radiomics, genomics, proteomics, and metabolomics, focusing on how AI leverages the deep mining of high-dimensional features to construct a non-invasive, high-sensitivity biomarker framework. This framework is designed to provide precise decision support for initial clinical screening and the critical differentiation between benign and malignant pulmonary nodules.

### AI in radiomics

The early and precise identification of LC is critical if we are to improve patient outcomes [58]. Although low-dose computed tomography (LDCT) has improved detection rates, this technology remains limited by low sensitivity for minute lesions and high false-positive rates [59–62]. Therefore, there is an urgent need to develop non-invasive strategies that can effectively identify suspicious lesions and enhance diagnostic reliability.

Driven by rapid advancements in AI technology, computer-aided diagnosis (CAD) has evolved from traditional image analytic methods to a deep learning paradigm centered on CNNs [63–65]. These advanced frameworks are capable of automated lung nodule detection and risk stratification, thereby significantly enhancing the level of automation and efficacy of screening procedures [66, 67]. For instance, Hung et al. developed a highly accurate machine learning (ML) model based on radiomic features, demonstrating potential applicability for assessing the risk of malignancy for indeterminate pulmonary nodules (IPNS) detected during screening [68]. More importantly, through the deep integration of deep learning and radiomics, Jacobs et al. revealed a pattern in which model performance for predicting lung nodule malignancy exhibited a “rapid ascent followed by a plateau” as the scale of data increased [68]. Moreover, Jacobs et al. demonstrated that only 20% of the training data was required to achieve a diagnostic performance comparable to that of clinicians [68]. This finding not only emphasizes the advantages of AI in resolving complex imaging features but also suggests that future breakthroughs in medical imaging AI should shift focus from the mere pursuit of data scale to deep strategies emphasizing data diversity and the coverage of complex cases [68].

In recent years, AI models have been driving a paradigm shift in screening from singular “nodule detection” to “long-term risk quantification” [69, 70]. For example, an externally validated deep learning algorithm, the LCP-CNN, can predict the malignancy of current pulmonary nodules using LDCT images, thus allowing for the prioritization of patients with suspicious nodules while reducing screening intensity for those with low-risk nodules [70]. Similarly, the ScreenLungNet deep learning model integrates features of whole-lung architecture, inflammatory background, and multiple nodules to generate an individualized 3-year LC risk score based solely on a single baseline LDCT scan [69]. In the NLST cohort, ScreenLungNet demonstrated high diagnostic accuracy (94.8%) and an AUC of 0.93 (95% CI: 0.92, 0.94) [69]. To better evaluate its clinical utility, the model achieved a sensitivity of 84.9% and a specificity of 95.2% [69]. Furthermore, it reported a positive predictive value (PPV) of 44.0% and an exceptionally high negative predictive value (NPV) of 99.3%, underscoring its reliability in excluding low-risk individuals [69]. This breakthrough elevates screening from a binary assessment to a continuous risk quantification tool, thus enabling the provision of precise recommendations for follow-up intervals and marking a substantial leap toward dynamic risk management [69]. Furthermore, studies utilizing the PanCan risk model and various deep learning algorithms have confirmed that AI possesses robust discrimination and calibration capabilities to evaluate the risk of malignancy for nodules, achieving performance levels comparable to those of clinical experts [71, 72].

Overall, the deep integration of AI and medical imaging is transforming LC screening from mere image interpretation into a comprehensive assessment system that combines anatomic feature analysis and long-term risk prediction. Radiomics-based AI models are capable of efficiently processing large-scale imaging data and precisely quantifying lesion morphology, density, and growth patterns, thereby significantly enhancing diagnostic efficiency and reducing the risk of missed detection. In the future, through multicenter and prospective validation, and continuous improvement in algorithmic generalization capabilities, AI is expected to become an indispensable intelligent tool for the early detection, precise intervention, and risk-stratified management of LC.

### AI in genomics

Over the past decade, genome-wide association studies (GWAS) have successfully identified numerous genetic susceptibility loci associated with LC, thus providing critical insights into the genetic basis of this disease [73–75]. However, the application of these findings in clinical practice remains limited thus far, primarily because individual susceptibility loci typically account for only a small fraction of the overall genetic risk [76–78].

Genetic assessment for LC is undergoing a paradigm shift from “single-gene analysis” to the integrative approach of polygenic risk scores (PRS) [74, 79–82]. By integrating and weighting the vast number of susceptibility loci identified by GWAS, PRS can more comprehensively quantify a specific individual’s genetic background [83]. For instance, sequence-based deep learning models such as “DeepSEA” or “Enformer” can directly predict the functional impact of variants from DNA sequences. These models can help GWAS to prioritize functional loci, thereby enhancing the biological interpretability and predictive power of PRS [83].

With the deep integration of next-generation sequencing (NGS) and ML, cell-free DNA (cfDNA) analysis has transcended the limitations of single-gene mutation detection, shifting toward the integrative analysis of multi-dimensional features [84, 85]. This shift has significantly improved both the sensitivity and specificity of early LC detection [84, 85]. Addressing the dual challenges of sensitivity and specificity in early diagnosis, AI models that are based on cfDNA fragmentomics have achieved pioneering breakthroughs [86]. Researchers proposed a multi-modal feature fusion strategy using a logistic regression algorithm to combine cfDNA end motifs with fragment size [86]. By decoding tumor-specific nuclease cleavage patterns, this model significantly enhanced the detection rate of stage I LC [86].

To address the clinically intractable challenge of differentiating malignant from benign pulmonary nodules, Wu et al. introduced a robust Ensemble learning framework [85]. Employing a stacking strategy, Wu et al. integrated independent predictions of fragmentomic features from multiple base classifiers, such as Support Vector Machines (SVM) and Random Forests. This approach substantially improved the specificity for excluding benign nodules while maintaining high sensitivity, effectively reducing the risk of over-diagnosis [85]. In addition to mining physical cfDNA features, AI has demonstrated transformative potential for deciphering epigenetic modifications in cfDNA [84, 87]. For the early screening of non-small cell lung cancer (NSCLC), researchers focused on the epigenetic marker 5-hydroxymethylcytosine (5hmC) and developed an SVM-based diagnostic model [84]. This study demonstrated that AI could identify specific 5hmC modification profiles in cfDNA, thus enabling precise LC screening at early stages in which mutation abundance is extremely low [84]. Furthermore, addressing the bottleneck in the clinical implementation of small cell lung cancer (SCLC) transcriptomic subtyping caused by difficulties in sample acquisition and RNA degradation, researchers proposed a novel epigenetic subtyping strategy based on DNA methylation [87]. Utilizing a Random Forest algorithm, these authors constructed a high-precision ML classifier and successfully validated the conservation of these methylation features in cfDNA [87]. This achievement enabled non-invasive molecular subtyping and chemosensitivity prediction for SCLC using only blood samples, thus providing a robust and clinically translatable epigenetic tool for the precision management of SCLC [87].

Collectively, the genomics-based AI framework, driven by systematic integration and in-depth mining, has enabled the construction of risk prediction models for LC with higher precision, dynamic evolution, and multidimensional clinical operability. This advancement not only underpins the precise stratification of early screening but also bridges the gap between basic research and clinical application, thus providing a feasible pathway with which to translate genetic findings into precision prevention and control.

### AI in proteomics

By systematically integrating proteomic data by the application of ML algorithms, researchers have successfully constructed risk prediction models with prospective clinical value, thus establishing a new paradigm for non-invasive ultra-early screening of LC [88–92].

In the realm of plasma proteomics, Davies et al. utilized ML to screen complex protein profiles for key biomarker panels, achieving the identification of high-risk individuals 1 year or more prior to clinical diagnosis [88]. Even more significantly, in a large-scale prospective cohort study involving 52,913 participants, AI-driven analysis demonstrated not only superior early detection capabilities for NSCLC but also precise risk stratification years prior to clinical diagnosis [89]. regarding urine proteomics, researchers employing algorithms such as Random Forest identified a panel of five urinary protein markers, including FTL and RAB33B [93]. This composite model achieved an area under the curve (AUC) of 0.99 in distinguishing LC from healthy controls and effectively differentiated LC from other malignancies, thus demonstrating substantial potential for clinical translation [93].

Tumor-derived extracellular vesicles (EVs) are enriched with specific proteins [92]. By comparing the plasma EV proteomes of NSCLC patients with those of healthy individuals, researchers utilized LASSO regression to identify a seven-protein biomarker panel (including MVP and GYS1) that exhibited excellent diagnostic performance [94]. Furthermore, Xiao et al. employed AI to select five key proteins (e.g., TUBB3, RPS7) from 38 exosomal markers to construct a diagnostic model [90]. This model effectively distinguished benign nodules from lung squamous cell carcinoma, with the efficacy of specific proteins further validated by Enzyme-Linked Immunosorbent Assay (ELISA) [90].

Collectively, the deep integration of proteomics and AI has successfully mined a series of high-value biomarkers through the systematic analysis of expression profiles in blood, urine, and exosomes. This strategy has significantly enhanced capabilities for early diagnosis, risk stratification, and the differential diagnosis of challenging cases in LC. With the continuous optimization of detection technologies and the advancement of multi-center clinical validation, this non-invasive and precise intelligent analytical approach is poised to become a core tool in LC screening and clinical diagnostic systems, providing robust technical support for improving patient prognoses.

### AI in metabolomics

In this section, we focus on the use of metabolomic signatures as non-invasive, high-sensitivity biomarkers for initial screening and the differentiation of benign from malignant lesions. Through the systematic analysis of small-molecule metabolites in blood, metabolomics can directly reflect the pathophysiological phenotypes of a given organism [95].

The advent of AI, particularly deep learning, has made the direct processing of raw mass spectrometry signals feasible [96]. For instance, the deep learning framework DeepMSProfiler, by directly analyzing raw data, achieved high-precision differentiation between lung adenocarcinoma and benign nodules (AUC 0.99) in cross-center, large-scale studies, effectively overcoming challenges associated with batch effects and the interference of unknown metabolites [96]. In terms of clinical translation, AI models have demonstrated exceptional performance for non-invasive early screening [97]. A previous study reported that ML algorithms can effectively decode serum metabolic fingerprints, achieving the high-sensitivity prediction of early-stage lung adenocarcinoma by identifying specific metabolic patterns [97]. Furthermore, emerging graph neural network frameworks, such as M-GNN, integrate multi-source information by constructing heterogeneous graphs [98]. This approach not only achieves high levels of diagnostic accuracy but also enhances model interpretability by identifying key metabolic nodes (e.g., choline and valine), thereby revealing deep associations between smoking, metabolic disorders, and the progression of LC [98].

Overall, the AI-driven systematic intelligent analysis of metabolomics is poised to become a transformative tool for non-invasive ultra-early screening and the diagnosis of LC.

## Application of artificial intelligence in prognosis assessment and drug sensitivity prediction for LC

While the previous chapter detailed diagnostic applications, this section evaluates AI’s role in post-diagnosis clinical management, including survival prediction and therapeutic response monitoring.

### AI in pathomics

Pathomics has endowed microscopic morphology with the attributes of big data by transforming unstructured tissue section images into vast, mineable, high-dimensional digital datasets, thereby establishing a standardized data foundation for AI-driven analysis [99, 100].

In the realm of LC, AI-based pathological image analysis has demonstrated exceptional clinical utility [101]. Deep learning algorithms are capable not only of automatically identifying morphological features that are closely associated with prognosis to successfully stratify patients with distinct survival outcomes achieving predictive efficacy (with an AUC up to 0.97) comparable to that of senior pathologists but also of establishing a profound link between microscopic morphology and molecular characteristics [101, 102]. Furthermore, a recent breakthrough involved the development of an annotation-free and deep learning-driven AI tool, the deep learning-enabled GEne mutation predictor (DeepGEM), which can predict gene mutations from routinely acquired histological slides [102]. Collectively, these models represent a highly promising ancillary tool for guiding the clinical management of patients with LC [101, 102].

For SCLC, a highly aggressive subtype, AI has similarly unveiled heterogeneity that remains elusive to conventional methodologies [103, 104]. Utilizing an unsupervised deep representation learning model, Zhang et al. performed high-resolution analysis on whole-slide images (WSIs) derived from 517 SCLC patients, and identified two histomorphological subtypes with significant biological distinctions: HIPOS-I and HIPOS-II [103]. The HIPOS-I subtype is characterized by robust immune cell infiltration and is associated with prolonged overall survival (OS) and disease-free survival (DFS) [103]. Conversely, the HIPOS-II subtype manifests with increased fibrosis, cellular pleomorphism, and dysregulated oxidative phosphorylation metabolism; these characteristics are indicative of a poor prognosis [103]. Furthermore, models based on dual-channel GNNs, such as DeepTFtyper, integrate information from the TME to predict transcription factor-based molecular subtypes directly from routine histological slides [104].

In the context of predicting therapeutic efficacy for immune checkpoint blockade (ICB), AI-driven quantitative scoring of immunohistochemical PD-L1 expression in tumor tissues analyzed via WSIs stained by 22C3 pharmDx has demonstrated the capacity to accurately predict tumor response and progression-free survival (PFS) in patients with advanced NSCLC [105]. Importantly, the superior accuracy of this technique compared to conventional pathological scoring has been validated in clinical specimens [105].

Overall, through training on massive datasets of WSIs, AI is capable of automatically decoding sub-visual morphological patterns that are imperceptible to the human eye, including nuclear arrangement, textural heterogeneity, spatial structural topology, and patterns of stromal and immune infiltration within the microenvironment. The deep integration of pathomics and AI is creating a new era in CAD and prognostic prediction for LC that is characterized by quantitative imaging, reproducible outcomes, and highly integrated information.

### AI in single-cell RNA sequencing

Single-cell RNA sequencing (scRNA-seq) offers an unprecedented single-cell perspective for dissecting the TME of LC, but faces challenges arising from high noise and data sparsity [6, 106–108]. AI overcomes these limitations by applying exceptional pattern recognition capabilities, significantly enhancing the depth of data mining [109]. This “AI + single-cell omics” paradigm is reshaping our understanding of the TME and empowering precision medicine [17, 110]

The advantage of integrating AI with scRNA-seq lies in its ability to robustly extract biological features that are directly associated with specific clinical phenotypes (e.g., prognosis and therapeutic resistance) from the inherent noise of scRNA-seq data [111]. For instance, Deep scSTAR, a deep learning-based framework, was developed to tackle the high sparsity and noise in single cells [111]. By mapping high-dimensional expression profiles into a robust latent feature space, this framework successfully identified a rare HSP+, FKBP4+ and CD8+ T cell subpopulation (approximately 8.08% of the total CD8+ T cell population) in NSCLC that was associated with resistance to immunotherapy, thus demonstrating the power of AI for decoding complex TME phenotypes [111].

To address the challenge of linking cellular heterogeneity with clinical outcomes, scBGDL employs a hybrid architecture combining GATs and MinCutPool layers to integrate scRNA-seq and bulk transcriptomic data, effectively identifying robust clinical cancer subtypes [112]. Furthermore, this technique provides a new tool for prognostic prediction and the optimization of treatment strategies [112]. Multiple studies have utilized ML algorithms, such as LASSO and Random Forest, to translate key cell subpopulation features identified in single-cell data, including cancer-associated fibroblasts, dendritic cells, macrophages, and B cells, into gene signatures that are applicable to bulk sequencing data [113–117]. These gene signatures have demonstrated excellent capabilities for prognostic assessment and the prediction of immunotherapy responses across multiple independent cohorts [113–117].

To decode the complex remodeling of the immune microenvironment during lymph node metastasis, Zhang et al. employed an ensemble ML approach [118]. By integrating single-cell derived signatures—specifically SPP1+ macrophage markers—with bulk transcriptomics, the authors constructed a robust LNRScore. The prognostic value of this model was validated in 493 samples, where it demonstrated high predictive accuracy for lymph node metastasis with an AUC of 0.80 [118]. Furthermore, the LNRScore was able to effectively predict the response to immunotherapy (TCIA), thus facilitating individualized risk stratification and clinical decision-making for patients with LUAD [118]. Furthermore, integrative analyses, combining single-cell transcriptomics and ML to investigate ac4C RNA modifications and m6A-related programmed cell death genes, have defined novel prognostic models, thereby linking epigenetic regulation to cell fate [119, 120]. Surprisingly, to overcome the limitations of single-model selection bias, studies have shifted toward comprehensive ML benchmarking [121]. For example, Cheng et al. systematically evaluated 26 ML algorithms (including DeepSurv, RSF, and LASSO) and various combinations of these algorithms [121]. By identifying an optimal algorithm pair by rigorous validation, these authors established an AI network-guided signature, setting a new standard for the robustness and generalizability of prognostic models in lung adenocarcinoma [121].

In summary, the deep integration of AI and scRNA-seq is systematically dissecting the complexity of the TME in LC. By identifying key functional subpopulations in a precise manner, decoding deep metabolic and epigenetic features, and characterizing spatial heterogeneity, this interdisciplinary field not only enhances our understanding of LC biology but has also identified novel biomarkers and therapeutic targets for clinical translation. With the continuous optimization of algorithms and the advancement of prospective clinical validation, AI-driven single-cell analysis is poised to become a core engine for achieving personalized precision medicine in LC.

### AI in spatial transcriptomics

Spatial transcriptomics (ST) enables the comprehensive profiling of gene expression while preserving in situ spatial coordinates, effectively integrating “tissue positional information” with “molecular transcriptomic data” [122, 123].

With the deep coupling of ST and AI, we have gained a novel perspective for revealing the complexity of the TME [124–126]. Initially, at the mechanistic level, the synergistic application of ML and spatial analysis allows for the deep deconvolution of the complex TME [124]. By identifying inter-cellular communication patterns and spatially distinct pathways of apoptosis, this approach has elucidated the intrinsic logic governing tumor survival and progression [124]. Moreover, the profound convergence of AI and ST exhibits remarkable translational potential for clinical management [125]. In immunotherapy, AI-powered spatial analysis can quantify the density and topological architecture of tumor-infiltrating lymphocytes (TILs), thus identifying robust spatial biomarkers that complement traditional methods for assessing the efficacy of immune checkpoint inhibitors (ICIs) [125]. Furthermore, with regards to acquired resistance to EGFR-TKIs, the integration of AI and spatial technologies facilitates the dynamic tracking of extensive TME remodeling, specifically the compositional and spatial evolution of TILs and fibroblasts [126]. Models derived from these spatial signatures have demonstrated significant predictive value for ICI response, thereby guiding decision-making for second-line therapies following the acquisition of resistance [126].

Collectively, the profound convergence of AI and ST is systematically unraveling the spatial organizational laws and functional logic of LC. In addition to refining our mechanistic understanding of tumor heterogeneity, evolution, immune evasion, and drug resistance, this cross-disciplinary frontier is critically transforming these biological insights into actionable, quantifiable spatial biomarkers and predictive clinical models. This advancement opens new avenues for the improved treatment of LC.

### AI in metabolomics

Building upon the discussion of AI-driven metabolomics in early screening and diagnosis, this section evaluates its clinical utility as a dynamic indicator for prognostic stratification and real-time therapeutic monitoring. In contrast to its static role as a diagnostic biomarker in early-stage detection, metabolomics in this context functions as a longitudinal tool, enabling AI to capture evolving metabolic signatures during treatment interventions.

Metabolomics unveils the functional state and intrinsic activities of tumors, serving as a critical window for understanding their growth, spread, and treatment response [95, 127, 128]. The AI-driven analysis of metabolomics may provide evidence for prognostic assessment and treatment decision-making in LC [129–131].

AI exhibits powerful data mining capabilities for prognostic stratification [129]. A previous study demonstrated that analyzing metabolomic profiles from tumor core biopsies via an ensemble ML strategy could effectively overcome single-algorithm biases, thus allowing for the construction of robust prediction models for survival [129]. In terms of molecular subtyping and therapeutic guidance, Bednarz et al. employed histology-guided spatial metabolomics combined with unsupervised ML algorithms (e.g., clustering analysis) to successfully perform precise metabolic subtyping across the tumor core, stroma, and interface regions of NSCLC [132]. This study not only validated established metabolic differences between adenocarcinoma and squamous cell carcinoma but, crucially, identified a rare isocitrate dehydrogenase (IDH)-mutant NSCLC subgroup [132]. This finding provided a novel molecular basis for developing individualized treatment strategies that target specific metabolic vulnerabilities [132]. Furthermore, for advanced LUSC, metabolomics-based ML models have been proven to accurately predict the efficacy of chemoimmunotherapy [130]. Simultaneously, in the comprehensive management of radiation therapy, AI-driven metabolic analysis successfully identified diagnostic and prognostic biomarkers that were capable of monitoring radiotherapeutic response by capturing dynamic metabolic fingerprint changes pre- and post-treatment [131].

In summary, from static survival prediction to the dynamic monitoring of chemoradiotherapy and immunotherapy, the fusion of AI and metabolomics is translating microscopic metabolic molecules into valuable quantifiable tools to support clinical decision making.

## Clinical application of artificial intelligence-driven multimodal data analysis in LC

**Fig. 2:**
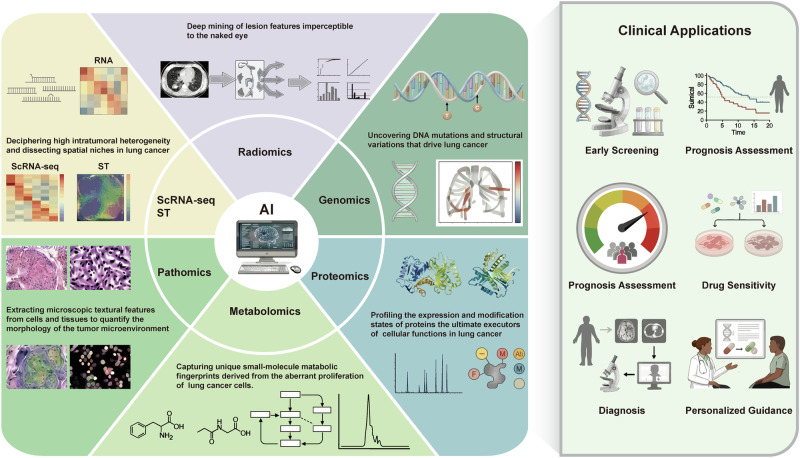
The landscape of AI-driven multi-omics integration for precision oncology in LC. The left hemisphere depicts the comprehensive data ecosystem fed into the AI engine, encompassing six key dimensions: (1) Radiomics: Deep mining of sub-visual imaging features; (2) Genomics: Identification of driver mutations and structural variations; (3) Proteomics: Profiling of protein expression and modification states; (4) Metabolomics: Capture of small-molecule metabolic fingerprints; (5) Pathomics: Quantification of tumor microenvironment morphology; and (6) scRNA-seq & ST: Dissection of intratumoral heterogeneity and spatial niches. The right panel highlights the translational clinical applications derived from this multi-dimensional synthesis. By integrating these heterogeneous datasets, AI models facilitate early screening, prognosis assessment, risk stratification, drug sensitivity prediction, precise diagnosis, and the formulation of personalized guidance for clinical decision-making.

AI-based multi-omics analysis is currently reshaping diagnostic and therapeutic paradigms for precision medicine in LC, progressively driving screening, diagnosis, and treatment strategies toward high levels of individualization and precision (Fig. 2) [22, 47, 133]. Current research regarding AI models involving multimodal data integration in LC is summarized in Table 2.

**Table 2:** Multi-omics-based artificial intelligence clinical application in LC.

AI Category	Multi-omics	Clinical application	Dataset	Study design	XAI implementation methodology	Performance benchmark	References
Machine learning	Radiomic and whole-genome sequencing	Risk-stratified classification of pulmonary nodule malignancy	Multi-center 1356 patients	Retrospective	SHapley Additive exPlanations	AUC 0.95	[134]
Machine learning	Radiomic and whole-genome sequencing	Accurate classification of pulmonary nodules	Multi-center 839 patients	Prospective	Multimodal Feature Attribution	AUC 0.85	[138]
Machine learning	Radiomic and whole-genome sequencing	Risk stratification of pulmonary nodules	Multi-center 963 patients	Retrospective	Decision Curve Analysis	AUC 0.81	[137]
Logistic regression algorithm	Proteomics and metabolomics	Enhance the diagnostic accuracy	318 patients	Retrospective	SHapley Additive exPlanations and Feature Importance	AUC 0.96	[135]
Generalized linear model (GLM) and machine learning	Radiomics and Genomics	Predicting the prognosis	151 patients	Retrospective	Image Biomarker Standardization Initiative/Generalized Linear Models	AUC 0.87	[140]
Machine learning	Single-cell RNA sequencing and spatial transcriptomics.	Predicting the prognosis	GEO and TCGA	Retrospective	Mechanism-based Interpretability/Mendelian Randomization	-	[124]
Deep learning	Pathomics and genomics	Prediction of cancer survival	TCGA + CPTAC + NLST	Retrospective	Graph Attention Mechanism	AUC 0.64	[56]
Multi-modal Deep Learning	Pathomics and transcriptomics	Predicting the prognosis	TCGA	Retrospective	Feature Mapping	-	[13]
Machine learning	Histology, spatial cellomics and multiplex immunofluorescence imaging	Enhances risk stratification	1168 patients	Retrospective	Interpretability-by-design	-	[208]
Supervised learning	Integrates WSIs (whole-slide images) with dense clinical data	Predicting the Prognosis	618 patients	Retrospective	Attention Heatmaps/Feature Importance	AUC 0.8	[326]
Machine learning	genomics and Single-cell RNA sequencing	Guidance for personalized treatment	1231 patients	Retrospective	Mechanistic Interpretability/Multi-dimensional feature attribution	AUC 0.65	[155]
Machine learning	Single-cell RNA sequencing and spatial transcriptomics.	Assessing prognosis and guiding personalized treatment	TCGA and GEO	Retrospective	Post-hoc biological interpretation	-	[153]
Deep learning	Single-cell RNA sequencing and spatial transcriptomics data	Predicting treatment response	-	Retrospective	Feature Scoring and Attribution	-	[111]
Machine learning	Single-cell RNA sequencing and spatial transcriptomics data	Personalized cancer immunotherapy prediction.	TCGA	Retrospective	SHapley Additive exPlanations/Genetic Algorithm	AUC 0.76	[154]
Machine learning	Pathomics, radiomic and transcriptomics	Prediction for first-line immunotherapy outcome	317 patients	Retrospective	Attention Heatmaps	AUC 0.81	[51]
Transfer learning	Radiomics and clinical information	Prediction of EGFR mutation	516 patients	Retrospective	Gradient-weighted Class Activation Mapping	AUC 0.883	[156]
Deep learning	Radiomic and pathomics,	Accurately classifying EGFR mutations	396 patients	Retrospective	Feature Interaction-Guided Fusion/Decision Curve Analysis	AUC 0.85	[53]
Deep learning	Radiomic and pathomics,	Pulmonary adenocarcinoma grade	988 patients	Retrospective	Ensemble Learning Attribution	AUC 0.92	[141]
Deep learning	Radiomics and genomics	Predicting EGFR genotype and targeted therapy response	18,232 patients	Retrospective	Feature Attribution/Gradient-weighted Class Activation Mapping	AUC 0.81	[159]
Deep learning	Clinico-genomics data	Evaluating the effectiveness of cancer treatments in relation to specific tumor Mutations	78,287 patients	Retrospective	Feature Attribution	AUC 0.7	[157]
Machine learning	Proteomics-Empowered Microfluidic-SERS Immunoassay	Guidance for personalized treatment	6 patients	Retrospective	Feature Importance & Selection	AUC 0.81	[161]
Machine learning	Next-generation sequencing (NGS) and multiplex immunohistochemistry (mIHC)	Guidance for personalized treatment	257 patients	Retrospective	Spatial Feature Engineering	AUC 0.83	[162]
Deep learning	Pathomics and genomics	Predicting NSCLC surgical outcomes	337 patients	Retrospective	Attention/Heatmap	AUC 0.99	[164]
Integrative Machine LASSO and Stepwise Cox	Single-cell RNA sequencing and metabolomics	Predicting the prognosis of patients	TCGA and GEO	Retrospective	Biological Feature Engineering	AUC 0.74	[144]

### Early screening and diagnosis

Emerging multi-modal AI integration strategies are circumventing the limitations of single data sources, opening a transformative path for enhancing the efficacy and accuracy of early LC detection [134–136].

The translational value of these tools lies in their ability to resolve the diagnostic ambiguity of IPNs [134]. For example, Liang et al. aimed to construct non-invasive diagnostic tools that fused multi-dimensional information [134]. By developing a ML model that integrated LDCT radiomics with cfDNA fragmentomics, these authors achieved complementary integration of macroscopic imaging and microscopic molecular features [134]. Translational impact is demonstrated by the model’s ability to reclassify nodules that appear suspicious on imaging but are biologically inert, thereby drastically reducing unnecessary invasive biopsies and their associated costs and risks [134]. AI has further driven synergistic complementarity across broader dimensions [136]. By combining liquid biopsy data, deep learning-extracted CT imaging features, and clinical variables, numerous researchers have achieved cross-validation and synergistic enhancement of different types of modal information [136, 137]. For instance, integrated models, such as PulmoSeek Plus, can fuse clinical characteristics, imaging markers, and cell-free DNA methylation data, to achieve precise risk stratification for lung nodules, effectively avoiding unnecessary and invasive diagnostic techniques [137, 138]. Furthermore, comprehensive models based on the AI-integration of plasma proteins and metabolites have further validated their superior performance for the diagnosis of NSCLC [135].

In summary, AI-driven multi-modal integration strategies are establishing a new ecosystem of “radiomics + liquid biopsy” for non-invasive diagnosis. The core clinical value of this technique lies in a dual benefit: on one hand, precisely eliminating biologically inert benign nodules to drastically reduce the risk of overtreatment; and on the other hand, significantly enhancing the detection rate for malignancies. As multi-omics sequencing costs decrease and AI algorithms evolve, these digital diagnostic tools, which integrate non-invasiveness, dynamic monitoring, and panoramic profiling, are poised to reshape the screening pathway for LC and serve as a key engine for advancing the frontiers of precision medicine.

### Prognostic prediction and patient risk stratification

The accurate prediction of survival outcomes and risk of recurrence serves as the cornerstone for achieving personalized and full-cycle management of LC [139]. Currently, AI-driven multi-modal data fusion strategies are significantly advancing prognostic stratification in LC toward greater precision by integrating biological information across distinct dimensions [13, 56, 140–142].

At the level of macroscopic non-invasive assessment, the integration of radiomics and genomics has opened significant new avenues [140, 142]. Addressing the limitation that single-modality imaging cannot fully reflect molecular heterogeneity, previous studies have indicated that “radiogenomic” dual-modal strategies constructed by Generalized Linear Models and ML algorithms can effectively extract deep radiomic features from CT images and complement these with genomic information, thereby achieving high-precision prediction for the risk of tumor recurrence [140, 142]. In addition, Yang et al. developed and validated an integrated learning model with both high accuracy and biological interpretability for predicting the pathological grade of invasive LUAD [141]. These authors integrated the macroscopic and microscopic features of pre-treatment CT images and postoperative full-field digital pathological sections to construct a radiopathological omics model (DHRPs) that combined deep learning and manual features [141]. This model not only demonstrated excellent diagnostic performance in [13] the correlations between imaging and pathological features, including cell proliferation, metabolic reprogramming, and changes in the immune microenvironment [141].

The deep fusion of pathological images and transcriptomic data has emerged as a key breakthrough for the elucidation of tissue heterogeneity [13]. Recent research has proposed a multi-modal analysis framework integrating computational pathology and multi-transcriptomics [13]. This approach utilizes deep learning to extract high-dimensional morphological features from WSIs and establishes cross-modal correlations with gene expression data via graph representation and attention mechanisms [13]. This image-text fusion strategy not only systematically characterizes the heterogeneity of LUAD from both morphological and molecular dimensions revealing intrinsic links between cell infiltration patterns in the tumor microenvironment and histological phenotypes, but also created a prognostic model that significantly outperforms single-modal approaches [13]. This highlights the clinical potential of combining cost-effective pathological data with deep molecular insights [13].

Furthermore, AI applications are expanding into microscopic mechanisms and multi-dimensional omics [111, 124]. By integrating single-cell and spatial transcriptomic data via ML, researchers have been able to extract clinically relevant deep features at the level of the cellular ecosystem. For instance, Li et al. utilized AI to reveal the mechanisms governing spatial interaction between programmed cell death and smooth muscle cells within the TME, subsequently developing a prognostic model featuring key genes [124]. Addressing the challenge of metastasis, researchers employed an Artificial Neural Network (ANN) framework to deeply integrate spatial multi-omics and single-cell data, systematically revealing the pivotal driving role of glycosylation modification in LUAD metastasis [143]. Combined with Mendelian randomization, this study not only established a causal link between glycosylation and metastasis but also constructed a high-precision ANN-based predictive model for identifying high-risk patients [143]. Moreover, AI has extended into metabolic reprogramming. By applying ML to mine single-cell data, researchers identified a taurine metabolism signature that can robustly predict prognosis and characterizes the Tumor Immune Microenvironment (TIME), thereby predicting potential responses to immunotherapy [144].

Therefore, AI-driven multi-modal fusion has created a panoramic prognostic assessment system by integrating macroscopic imaging, microscopic pathology, and deep molecular omics, thus proving a solid foundation for precise stratification and individualized treatment planning for LC.

### Prediction of immunotherapy efficacy and regimen optimization

Although the clinical application of ICIs marks a major breakthrough in the history of LC treatment, the clinical benefits of these drugs still face severe challenges in that significant inter-individual heterogeneity exists in terms of treatment response rates, and some patients face risks of immune-related adverse events (irAEs) [145, 146]. In this situation, the deep integration of AI and multi-modal data offers an innovative pathway for precision medicine by overcoming the spatiotemporal limitations of traditional tissue biopsies[147–149].

At the level of macroscopic non-invasive assessment, AI-constructed models of “digital biopsy” are redefining efficacy monitoring paradigms [148, 149]. These non-invasive tools not only achieve precise prediction of clinical outcomes such as immunotherapy response, pathological complete response (pCR), and immune-related pneumonitis but also demonstrate the potential to non-invasively assess PD-L1 expression, tumor mutational burden, and the TME status [148, 149]. For instance, with regards to neoadjuvant chemoimmunotherapy, multi-modal CT deep learning models have been shown to predict pCR in NSCLC in a highly efficient manner [150]

In addition to the assessment of efficacy, AI plays a pivotal role in predicting treatment-related toxicities, particularly in the complex clinical scenario of symptomatic pneumonitis induced by combined radiotherapy and immunotherapy [149]. Yang et al. proposed a prediction framework based on multimodal deep learning (MDL), innovatively fusing clinical characteristics, dosimetric parameters, and deep radiomic features from CT images [149]. The biological rationale for this approach lies in the synergistic interplay between radiation-induced cellular stress and immune-mediated inflammatory cascades [151, 152]. The MDL framework specifically captures these complexities by utilizing 3D convolutional layers to extract latent spatial heterogeneity from CT scans, reflecting early sub-clinical tissue damage [151, 152]. These radiological features are then integrated with systemic clinical data via an attention-based fusion mechanism, allowing the model to capture the non-linear interactions between local physical damage and systemic immune activation [151, 152]. Consequently, this model demonstrated a superior AUC of 0.922 (95% CI: 0.902–0.945), providing a robust tool for predicting pneumonitis risk with high precision [151, 152].

Given the complex tumor heterogeneity in advanced patients, single-dimensional biomarkers often fail to capture the full clinical status [51]. For instance, in a large-scale cohort study of 317 metastatic NSCLC patients, PD-L1 expression as a standalone biomarker yielded a low C-index of 0.54 (95% CI: 0.51–0.57) for predicting overall survival, and failed to significantly stratify survival outcomes even in high-expression subgroups (PD-L1 expression greater than 50%) (*P* = 0.44) [51]. This quantitative evidence highlights the inherent limitations of relying on a single biological axis for complex clinical predictions [51]. To overcome this, researchers constructed a four-dimensional comprehensive predictive model based on AI, integrating clinical phenotypes, pathological WSIs, CT radiomics, and transcriptomic data. AI algorithms successfully spanned the data gap between macroscopic imaging phenotypes and microscopic gene expression to capture hidden non-linear patterns of interaction across different modalities within the tumor ecosystem [51]. Analysis confirmed that this multi-modal integrated model, which achieved an AUC of 0.81 for 1-year mortality, significantly outperformed any single-modality method for predicting the objective response rate and PFS for first-line immunotherapy [51].

With regards to the elucidation of microscopic mechanisms, AI-empowered multi-omics analysis has significantly enhanced our understanding of tumor biology [111, 153, 154]. By integrating single-cell transcriptomics with ST data, AI models can decode spatial interaction networks between cells to accurately predict the efficacy of immunotherapy [111, 153, 154]. For example, a recent study utilized ML algorithms to construct a Post-Translational Modification Learning (PTML) signature model, which innovatively integrated single-cell transcriptomics, ST, and large-scale clinical transcriptomic data, translating latent glycosylation features into quantifiable prognostic indicators for the first time [155]. By cross-validating multi-modal data, this AI model not only precisely identified different subtypes of lung adenocarcinoma with specific PTM patterns but also identified B4GALT2 as a key spatial regulator mediating the immune exclusion of CD8+ T cells [155]. Clinical validation demonstrated that this AI scoring model consistently achieved the highest C-index when benchmarked against 98 previously established LUAD prognostic signatures across seven independent cohorts [155]. Furthermore, it yielded robust predictive accuracy with AUC values exceeding 0.65 for 1-, 3-, and 5-year survival, significantly outperforming existing biomarkers in predicting the efficacy of ICIs and effectively identifying patients most likely to benefit from immunotherapy [155].

In summary, AI-driven multi-modal integration strategies have generated a high-precision and efficacious prediction system by bridging macroscopic imaging phenotypes with microscopic molecular mechanisms. This system not only optimizes patient selection and regimen formulation for immunotherapy but also provides new perspectives for understanding the biological mechanisms of treatment resistance, thus propelling immunotherapy for LC toward a dynamic and personalized model of precision medicine.

### Driver gene mutation identification and targeted drug discovery

In the field of precision targeted therapy for LC, the deep integration of AI and multi-modal data has become a core engine driving the identification of mutations and targeted drug discovery [156–158]. By leveraging fully automated AI systems and multi-modal deep learning technologies, such as feature interaction-guided fusion and three-dimensional CNNs, researchers have achieved the efficient extraction of whole-lung phenotypic information directly from medical images [156–158]. This image-gene mapping strategy not only allows for the accurate assessment of mutation status in key driver genes such as EGFR but also provides precise predictions for the efficacy and prognosis of targeted drugs such as EGFR-TKIs [53, 156, 159, 160]. Within the realm of drug development, the combination of AI platforms with technologies such as cell membrane chromatography and proteomics, has generated an efficient drug screening system, successfully accelerating the discovery and development of novel EGFR antagonists [158]. Furthermore, with regards to the optimization of clinical strategy, by utilizing large-scale clinical genomics data spanning tens of thousands of patients, AI can systematically characterize specific mutation-treatment effects [157]. By decoding the heterogeneity in drug response patterns to different genetic variants, AI offers deep data-driven insights for the discovery of new targeted drugs and the optimized application of existing drugs [157].

Overall, the deep integration of AI with multimodal data may enable an intelligent closed-loop system that can proceed from the identification of mutations to optimization of the entire treatment strategy, thus providing strong technical support for guiding treatment with targeted drugs in a precise and scientific manner.

### Surgical guidance and complication prediction

The deep integration of AI and multimodal data is profoundly reshaping the decision-making process and risk management paradigm for the surgical treatment of LC, thus providing a new path for achieving precise and personalized diagnosis and treatment throughout the entire cycle from preoperative planning to postoperative management [161–163].

In the preoperative phase, Zhang et al. successfully achieved the preoperative precise classification of highly invasive micropapillary (MPP) lung adenocarcinoma by integrating AI, proteomics, and micro-nano sensing technologies [161]. This AI-driven analysis platform overcomes the limitations of traditional imaging and pathological evaluations, providing more precise biological information for surgical decisions and ensuring the optimization of the surgical resection range for high-risk patients [161]. Furthermore, ML integrating NGS and multiplex immunohistochemistry (mIHC) data can decipher unique patterns of lymph node metastasis [162]. By predicting metastatic probability, these models provide an evidence base for non-surgical or individualized treatment regimens [162]. With regards to prognostic assessment, fully automated deep learning models based on histopathological images have demonstrated strong predictive capacity for the postoperative prognosis of patients with NSCLC [164].

In terms of postoperative management, ML models constructed from clinical data spanning tens of thousands of surgical LC patients can effectively predict the risks of postoperative complications, including cardiovascular and neurological events, thus enabling proactive risk management during the patient recovery process [163].

### Explainable AI: bridging the gap between the black box and clinical trust

The clinical adoption of AI in LC management is often hindered by the “black box” nature of deep learning models [98, 104]. To bridge the gap between technical validation and clinical trust, recent research has integrated Explainable AI (XAI) techniques to provide biological justifications for model predictions [98, 104].

In the M-GNN model, the identification of “key metabolic nodes” (such as choline and valine) is achieved through the following process: First, the model constructs a heterogeneous graph integrating metabolites, diseases, and biochemical pathways [98]. It utilizes a hierarchical structure of GraphSAGE and GAT to perform feature extraction and information propagation, placing originally isolated metabolite data within a complex biological network context [98]. Following model training, the researchers introduced the SHAP (SHapley Additive exPlanations) interpretation tool to quantify the contribution of each metabolic feature to LC prediction (i.e., SHAP values), thereby screening the Top 30 key biomarkers that most significantly influence classification decisions [98]. Choline (associated with cell membrane synthesis and tumor invasion) and valine (associated with the regulation of amino acid metabolism) were ultimately identified as key metabolic nodes with biological and clinical significance due to their prominent structural connectivity within the heterogeneous graph and their exceptionally high feature weights in the SHAP analysis [98].

DeepTFtyper employs the integrated gradients (IG) algorithm to calculate contribution scores for WSI patches, which are then visualized as spatial heatmaps overlaid on the original images [104]. To further bridge the gap between AI features and histology, the model utilizes the Leiden algorithm to cluster high-contribution patches, allowing for the identification of representative visual phenotypes associated with specific transcription factor subtypes [104].

A fundamental challenge remains the trade-off between model accuracy and interpretability. Complex architectures like deep neural networks or ensembles often achieve superior predictive performance by capturing intricate, non-linear relationships, yet their internal logic is difficult to parse (the “Black Box” problem). Conversely, intrinsically interpretable models, such as decision trees or linear regressions, offer transparency at the cost of capturing high-dimensional biological complexity. To address this, current LC AI research increasingly adopts post-hoc explanation tools (e.g., SHAP and IG) [98, 104]. These tools allow developers to maintain the high accuracy of complex models while providing clinicians with retrospective visual or quantitative evidence to justify clinical decisions [98, 104].

### Strengthening the translational pipeline: from model output to clinical action

The ultimate translational success of AI in oncology depends on its seamless integration into the multidisciplinary team (MDT) workflow [165–168]. Rather than providing isolated “black-box” scores, AI tools must offer explainable insights that clinicians can incorporate into treatment planning. For example, AI-driven radiomics can provide quantitative evidence of intratumoral heterogeneity, helping MDTs decide between aggressive surgical intervention and conservative monitoring for sub-solid nodules [165–168].

A key metric for translational impact is the reduction in time-to-treatment (TTT) [169–171]. AI platforms that automate routine tasks—such as lung nodule segmentation, response evaluation criteria in solid tumors (RECIST) 1.1 measurement, and automated reporting—allow radiologists to prioritize high-risk cases [169–171]. Prospective data suggest that AI-assisted triage can significantly shorten the diagnostic window, potentially preventing tumor progression during the wait-time for definitive therapy [165–167]. Translational impact is also realized through software-as-a-medical-device (SaMD) deployment in resource-limited settings. By providing expert-level diagnostic capabilities to community hospitals via cloud-based AI, we can standardize the quality of care, ensuring that rural patients receive the same early-detection benefits as those in specialized oncology centers [165, 166].

## Artificial intelligence and digital health: reshaping the new paradigm of full-cycle LC management and remote monitoring

Digital health (eHealth), representing the deep fusion of technology and medicine, marks the arrival of the era of high-performance medicine [165, 172]. In the management of LC, this integrated approach is fundamentally reshaping the paradigms of prevention, treatment, and rehabilitation through wearable devices equipped with AI algorithms, mobile health applications, and telemedicine services [173].

At the level of clinical practice, digital health tools utilize passive sensing technology to transform physiological data acquired from patients into clinically significant digital biomarkers [172]. Research indicates that data collected from consumer-grade smartphones and wearable sensors can objectively reflect a patient’s functional status, symptom burden, and risk of adverse clinical outcomes [172]. For instance, Li et al. reported a randomized controlled trial involving early NSCLC survivors, which confirmed the superiority of the home-based cardiac tumor rehabilitation model based on digital therapy (DTx) in improving the cardio-pulmonary fitness and quality-of-life of patients [174]. This study utilized a three-tier personalization algorithm to generate a 5-month AI-driven exercise prescription [174]. Initially, the algorithm calculated a target heart rate (HR) zone for each patient using the karvonen formula (HRresting + [HRmax − HRresting] × [≈40–60%])—based on baseline cardiopulmonary exercise testing (CPET) [174]. Throughout the intervention, the AI engine integrated real-time HR data from wearable devices to monitor “effective exercise duration” within this target zone and implemented a stepped-titration logic; this allowed for the dynamic adjustment of exercise duration (90–150 min/wk) and resistance intensity based on accumulated performance data and patient feedback [174]. Consequently, the peak oxygen uptake of patients in the intervention group increased significantly compared to the conventional care group, demonstrating high compliance and good safety while significantly alleviating cancer-related fatigue and anxiety [174]. This result indicates that digital therapy can serve as an effective alternative to traditional centralized rehabilitation by providing a safe, convenient, and efficient new path for the management of cardiac health in postoperative LC patients [174].

During chemoradiotherapy for LC, baseline step count data captured by wearable devices can predict post-treatment clinical outcomes [175]. In patients with advanced NSCLC, low step counts are not only significantly associated with a decline in quality-of-life and the exacerbation of depression but have also emerged as an independent prognostic factor for patients with local advancement [176]. Furthermore, clinical trials by Basch et al. demonstrated that digital remote symptom monitoring based on patient-reported outcomes (PROs) not only improved a patient’s quality of life but also significantly prolonged OS [177]. The median OS was 31.2 months (95% CI, 24.5–39.6) in the PRO group compared to 26.0 months (95% CI, 22.1–30.9) in the usual care group (difference, 5 months; *P* = 0.03) [177]. In the multivariable model, the survival benefit remained statistically significant with a hazard ratio (HR) of 0.83 (95% CI, 0.70–0.99; *P* = 0.04), establishing the core status of this approach in best supportive care for oncology [177].

**Fig. 3:**
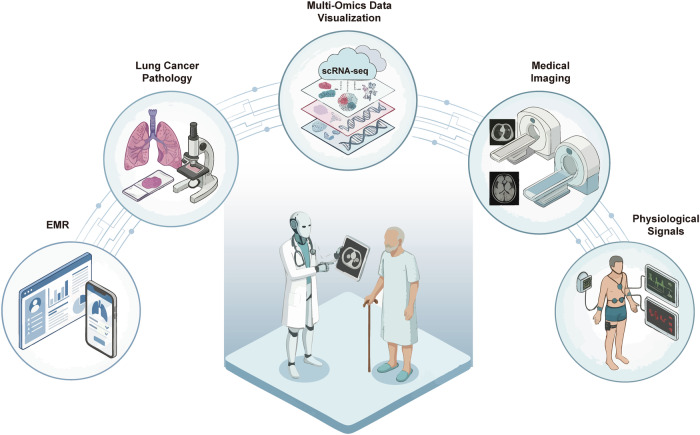
The ecosystem of AI-empowered multimodal data integration for holistic LC management. The schematic illustrates the convergence of five distinct data dimensions around the patient: (1) Electronic Medical Records (EMR): Comprehensive clinical history and health archives; (2) LC Pathology: Histological analysis and microscopic evaluation; (3) Multi-Omics Data Visualization: High-dimensional molecular profiling, including scRNA-seq; (4) Medical Imaging: Radiological data from CT and MRI scans; and (5) Physiological Signals: Real-time monitoring of vital signs. The central AI agent synthesizes these heterogeneous data streams to provide the patient with precise, full-cycle medical management and personalized decision support.

With the advancement of AI applications in precision medicine, future remote monitoring ecosystems are likely to extend beyond the tracking of vital signs to microscopic monitoring at the molecular level [178, 179]. An ideal scenario for the digital management of LC would involve patients wearing smart devices at home to quantitatively monitor physical functionality during rehabilitation, combined with miniaturized in vitro diagnostic devices, such as microfluidic-based liquid biopsy tools. AI could then be utilized to analyze circulating tumor components (e.g., ctDNA) and metabolites in biofluids in real-time. This dual monitoring mode of macroscopic vital signs and microscopic molecular profiles would enable comprehensive real-time management during the intervals between clinical visits, thereby facilitating proactive, holistic, and personalized interventions at the ultra-early stages of recurrence or metastasis (Fig. 3).

The profound integration of AI and digital health technologies is driving a shift in medical services from traditional in-hospital follow-up to home-based active monitoring. This intelligent and closed-loop digital management system transcends the spatiotemporal limitations of medical resources. This approach significantly enhances the quality of home-based rehabilitation for patients and provides solid technical support for precise early warning and timely intervention throughout the full cycle of LC. This heralds a broad prospect for the evolution of LC care toward a more intelligent, personalized, and globalized future.

## The challenges and limitations of artificial intelligence-driven multimodal data analysis in the clinical management of LC

**Fig. 4:**
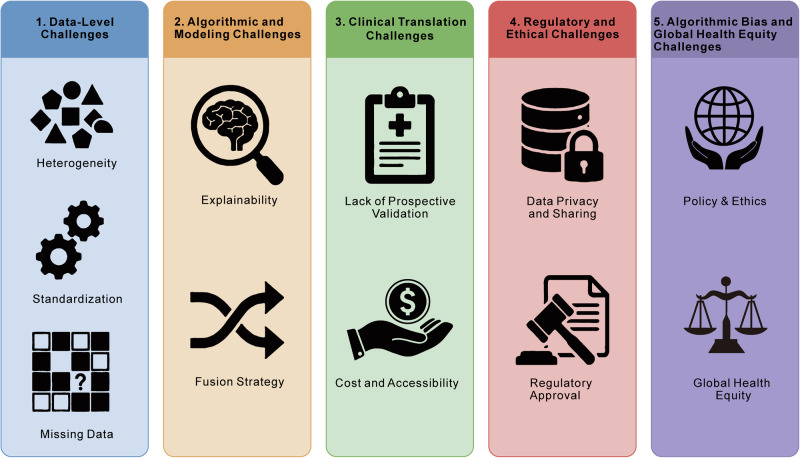
Major challenges hindering the clinical translation and widespread adoption of AI in LC. The diagram categorizes the obstacles into four distinct levels: (1) Data-Level Challenges: Issues stemming from the high heterogeneity of multi-source data, lack of standardization across institutions, and the prevalence of missing data in clinical datasets. (2) Algorithmic and Modeling Challenges: The difficulty in achieving model explainability (opening the “black box”) and developing robust fusion strategies for integrating multi-omics modalities. (3) Clinical Translation Challenges: The scarcity of large-scale prospective validation studies needed to prove clinical utility, alongside barriers related to implementation costs and accessibility in real-world settings. (4) Regulatory & Ethical Challenges: Critical concerns regarding patient data privacy, secure data sharing protocols, and the complexities of obtaining regulatory approval for AI-based medical devices. (5) Algorithmic Bias and Health Equity Challenges: The imperative to mitigate risks of AI-driven health disparities caused by unrepresentative data while establishing ethical frameworks to ensure technological advancements promote global health equity rather than widening the digital divide.

Although AI-driven multimodal data analysis has demonstrated immense transformative potential in the early screening, precise subtyping, prognosis, and treatment decision-making of LC, its translation from laboratory algorithms to widespread clinical tools still faces severe multi-dimensional challenges [180–184]. These limitations primarily concentrate on four core areas: the robustness of model performance, the complex heterogeneity of multi-omics data, social and ethical norms, and the level of clinical evidence (Fig. 4) [180–186].

### Data-level challenges

As precision medicine evolves toward multi-omics, the intrinsic heterogeneity of data has become a major technical barrier to the construction of AI models [187–191]. This can be summarized by three factors. First, challenges related to technical noise and standardization [187–191]. For example, different sequencing platforms, kits, and bioinformatics analysis pipelines introduce significant non-biological variations [187–191]. This technical noise directly interferes with the precise recognition of tumors by AI models, leading to poor consistency in cross-platform analyses [187–191]. Second, the spatiotemporal heterogeneity of LC, a disease that exhibits high intra-tumoral heterogeneity and undergoes clonal evolution over the course of treatment [9, 192, 193]. AI models trained solely on single-timepoint and single-site tissue biopsies or static sequencing data struggle to capture the dynamic mechanisms of drug resistance and microenvironmental remodeling, thus limiting their predictive accuracy for whole-course management [9, 192, 193]. Third, challenges in multimodal alignment. True precision diagnosis and treatment require the integration of multi-dimensional data, including genomics, transcriptomics, metabolomics, and radiomics [18, 194]. However, the scales, dimensions and noise patterns of different omics data vary widely. The lack of effective cross-modal alignment and fusion algorithms makes it very challenging to construct comprehensive models that reflect the biological characteristics of LC [18, 194].

To bridge this gap, emerging hierarchical fusion frameworks, such as the hierarchical multimodal co-attention transformer (HMCAT), have been developed to align multi-scale data [195]. HMCAT specifically tackles the vast difference in spatial scales between WSIs and radiology images by employing hierarchical radiology-guided co-attention mechanisms [195]. This approach allows the model to characterize complex multimodal interactions and align microscopic cellular features with macroscopic tissue phenotypes, consistently outperforming unimodal or simple fusion methods [195].

### Algorithmic and modeling challenges

The clinical efficacy of AI models depends not only on the accuracy of internal validation but, more crucially, on their robustness and transparency in the real world [165, 180, 185, 196]. However, existing medical data often exhibit a “siloed” distribution and lack unified standardization protocols [165, 180, 185, 196]. Models trained using data from a single center or specific equipment are highly prone to “overfitting”, thus leading to a significant decline in generalization ability during external validation or cross-center application, making it difficult for these models to adapt to the heterogeneous data environments of different medical institutions [165, 180, 185, 196]. With the increasing complexity of intermediate and attention-based fusion strategies, while enhancing predictive power, significantly contributes to the ‘black box’ dilemma by making it difficult to trace which specific biological features from which modality are driving the clinical output [52, 197–199].

The widespread clinical adoption of AI in oncology remains significantly hindered by the inherent “black box” nature of deep learning models [191, 200, 201]. This lack of transparency prevents clinicians from accessing interpretable diagnostic rationales, thereby restricting the depth of human-machine collaboration, particularly in high-stakes medical scenarios [191, 200, 201]. Furthermore, many existing AI models are optimized for single tasks and fail to effectively integrate multi-source heterogeneous data—such as electronic health records (EHRs), dynamic imaging, and longitudinal genomic sequences—making it difficult to seamlessly embed these tools into the complex clinical decision-making workflows managed by physicians [49, 185, 202, 203]. To dismantle this “black box” effect and enhance trust within the medical community, the development of XAI has become a critical direction for interdisciplinary research [204–207].

### Clinical translation challenges

Currently, the vast majority of AI research remains in the phase of retrospective study; this represents a major limiting factor with regards to regulatory approval and recommendation in clinical guidelines [101, 103, 126, 208]. Historical data is usually highly selected and curated but fails to fully simulate the complexity, confounding factors and increased frequency of missing data associated with real clinical scenarios [46]. When referring to retrospective datasets, high levels of accuracy do not equate to a genuine improvement in patient prognosis in future clinical practice.

#### External validation and real-world performance

A significant limitation of many current oncology AI models is their over-reliance on highly curated public datasets, such as TCGA and GEO, which may not capture the “noise” and complexity of routine clinical practice [209]. Ensuring generalizability requires rigorous external validation in independent, diverse cohorts [209].

Currently, fewer than 30% of published medical AI models undergo independent external validation in geographically or institutionally distinct cohorts [210–215]. This lack of rigorous testing often leads to “over-optimism” and a subsequent “performance drop”—typically an AUC decrease of 0.10–0.15—when models encounter “dataset shift” caused by diverse scanners, staining protocols, or patient demographics [180]. High-profile failures, such as the epic sepsis model and models that inadvertently “memorized” site-specific histopathology artifacts in TCGA data, underscore that superior internal performance does not guarantee real-world reliability [216–218].

To bridge the gap between technical precision and patient benefit, we must articulate how enhanced AI metrics, such as AUC and sensitivity, translate into meaningful clinical outcomes [139, 219, 220]. The primary value of AI in LC lies in driving a “stage shift”—facilitating diagnosis at earlier, resectable stages (I/II) rather than symptomatic, advanced stages (III/IV), where 5-year survival rates drop from 60–70% to below 10% [58, 221, 222]. Furthermore, superior diagnostic accuracy mitigates the “diagnostic odyssey” by providing precise malignancy risk scores. For instance, optellum, in collaboration with leading global universities and healthcare systems, has developed and clinically validated the virtual nodule clinic, an AI-powered LC diagnostic platform [99, 139, 168, 219, 220]. By leveraging AI-assisted image interpretation, the platform assists clinicians in differentiating between benign and malignant lung nodules, assessing malignancy risk levels, and providing longitudinal patient tracking. This integration significantly minimizes the necessity for invasive procedures, such as biopsies, in cases of benign lesions [100, 139, 168, 219, 220]. Ultimately, this precision-driven approach optimizes the therapeutic window, translating improved algorithmic performance into superior OS and quality of life (QoL) [139, 168, 223].

To bridge the gap between “in-silico” success and clinical reality, we propose a set of minimum standards for robust validation. This includes multi-site blinded testing across at least three independent centers and the inclusion of diverse data sources from multiple hardware vendors to ensure technical and demographic fairness. Furthermore, we advocate for prospective “shadow” testing, where models run on live clinical streams without influencing care to assess real-world behavior. Adherence to the transparent peporting of a multivariable prediction model for individual prognosis or diagnosis (TRIPOD) + AI reporting guidelines is essential to ensure transparency and reproducibility in these validation efforts, shifting the focus from mere statistical accuracy to genuine clinical generalizability [224].

#### The gap between technical validation and clinical benefit

To ground the discussion in current clinical realities, it is essential to acknowledge the AI tools that have already transcended retrospective validation to achieve regulatory clearance or enter prospective clinical evaluation [138, 225–228]. The landscape of LC diagnostics has been significantly shaped by FDA-cleared AI algorithms, primarily focusing on nodule detection and characterization [138, 225–228]. Tools such as AI-rad companion (siemens healthineers) have integrated AI to automate the identification of pulmonary nodules on CT scans [229, 230]. Virtual nodule clinic was the first AI clinical decision support software FDA-cleared for LC diagnosis [225, 231, 232]. It provides a “LC ikelihood” (LCL) score based on imaging features, helping clinicians manage IPNs more effectively [225, 231, 232]. Beyond imaging, the FDA has cleared AI-based tools like Paige Prostate (as a precedent in oncology) [233].

The shift toward “active intervention” is evidenced by several landmark prospective studies: (i) The NELSON and NLST Legacy: While these were screening trials, they paved the way for current randomized controlled trials (RCTs) evaluating AI integration [138, 234, 235]. (ii) AI in immunotherapy guidance: Prospective trials are now exploring the use of “radiomics-clinical” signatures to select patients for ICIs [138, 236–240]. An example includes the prospective validation of AI models to predict hyperprogressive disease (HPD), ensuring that high-risk patients are diverted to alternative therapies in real-time [138, 236–240].

Although the performance metrics of AI models in LC, such as AUC and C-index, have reached unprecedented levels in recent literature, a formidable “translational gap” persists between laboratory success and actual bedside benefit [138, 196, 241–245]. This disparity is primarily driven by three core contradictions [138, 196, 241–245].

(i) The disconnect between the “test effect” of retrospective data and real-world heterogeneity [245, 246]. The vast majority of AI models are developed using highly curated and standardized retrospective datasets [245, 246]. This “retrospective validation” is essentially equivalent to a model completing an “open-book exam,” often overlooking complex variables inherent in real-world clinical environments, such as disparate scanning protocols across institutions, equipment noise, and complex patient comorbidities. Consequently, superior performance on static datasets does not guarantee that a model will yield tangible survival benefits when faced with prospective, real-world patients.
(ii) The fundamental distinction between predictive accuracy and clinical utility. A high AUC value merely demonstrates the statistical discriminative power of a model, whereas true clinical benefit emphasizes the substantive optimization of diagnostic and therapeutic pathways [138, 241]. AI models must evolve from “passive observers” to “active intervenors.” For instance, the true clinical value of a model predicting immunotherapy response lies not in fitting historical data, but in demonstrating through RCTs—that decisions guided by the model significantly improve “hard” clinical endpoints, such as OS, PFS, or patient quality of life (QoL) [138, 241, 247].
(iii) The high barriers to prospective validation, which constrain interventional research. Conducting prospective RCTs entails substantial financial costs, rigorous ethical approvals, and years of clinical follow-up [242, 243]. As illustrated in Table 2, the majority of AI research in LC currently halts at retrospective external validation; very few studies have progressed to the interventional phase to evaluate the longitudinal impact of AI-assisted decision-making on patient prognosis.

In summary, a strategic shift in future research is imperative: moving from the singular pursuit of algorithmic precision on static datasets toward assessing the actual contribution of AI integration within clinical workflows to the longitudinal management of a patient’s entire life cycle.

#### Cost-effectiveness and implementation barriers

The clinical translation of multimodal AI systems is contingent not only on diagnostic accuracy but also on economic viability and equitable accessibility [248–251].

The total cost of integrating multimodal AI into hospital infrastructure is multifaceted, extending beyond the initial software procurement [249, 252, 253]. Key financial drivers include: (i) Data Integration and Interoperability: Consolidating heterogeneous data (e.g., genomics, radiology, and pathology) requires robust Middleware and Enterprise Imaging systems, often costing between $100,000 to $500,000 for mid-to-large scale hospitals; (ii) Computational Infrastructure: Running deep learning models on multi-terabyte datasets necessitates high-performance computing (HPC) clusters or GPU-accelerated cloud services, with ongoing operational costs; (iii) Personnel Training and Workflow Integration: The “hidden costs” involve training clinicians and IT staff to interpret and maintain AI-generated insights, which can account for 20–30% of the total implementation budget [249, 252, 253].

While initial costs are substantial, the long-term return on investment (ROI) is driven by clinical efficiency and personalized treatment:(i) Reduction in Unnecessary Procedures: AI-driven screening can reduce false-positive rates, potentially saving the healthcare system thousands of dollars per patient by avoiding unnecessary biopsies and follow-up scans. (ii) Early Intervention: By detecting LC at earlier stages (stage I/II), the system significantly reduces the high costs associated with treating advanced-stage disease and long-term hospitalizations. (iii) Operational Efficiency: Multimodal AI can automate the triage of complex cases, reducing the time pathologists and radiologists spend on routine quantification, thereby increasing departmental throughput.

To ensure that multimodal AI does not widen the healthcare gap, several strategies must be employed in resource-limited settings: (i) Cloud-Based “AI-as-a-service” (AIaaS): Utilizing cloud platforms allows smaller clinics in LMICs to access powerful models via a subscription or per-scan model, eliminating the need for expensive on-site hardware. (ii) Model Compression and Optimization: Developing “lightweight” models (e.g., through pruning and quantization) allows AI to run on existing, lower-specification diagnostic equipment. (iii) Public-Private Partnerships: Collaborations between governments and AI developers can subsidize implementation costs in underserved regions, treating AI infrastructure as a public health utility.

In summary, the clinical application of AI in the management of LC requires more than just the continuous iteration of algorithmic technologies to address “black box” and heterogeneity issues. It necessitates the establishment of standardized multi-omics shared databases to eliminate algorithmic bias and ensure global health equity. Furthermore, a commitment to rigorous and prospective clinical empirical research is essential to validate the clinical value and safety of AI in this setting. Ultimately, the sustainable integration of these systems depends on addressing the financial burden of implementation, ensuring that the economic feasibility and cost-effectiveness of multimodal AI are balanced to provide equitable access across both high-resource and resource-limited environments.

### Regulatory and ethical challenges

As AI technologies shift from fixed algorithms to continuously learning systems, regulatory frameworks are evolving to ensure safety without stifling innovation [254–258]. The traditional regulatory pathway for medical devices is ill-suited for AI/ML algorithms that improve over time [257–259]. In response, the FDA has proposed a total product lifecycle (TPLC) regulatory framework [257–259]. Central to this is the pre-market assurance of real-world performance (RWP), where manufacturers submit a pre-determined change control plan (PCCP) [257–259]. This plan outlines anticipated algorithmic modifications and the protocols used to implement them safely, allowing for rapid iterations while maintaining rigorous oversight [257–259].

A cornerstone of modern AI regulation is the shift toward continuous monitoring. Unlike static software, AI requires ongoing performance evaluation to detect “model drift” [257–259]. Regulatory bodies now advocate for post-market surveillance that includes “shadow mode” deployment—where the algorithm runs in parallel with clinical practice to collect real-world data without directly influencing patient care—until its stability and safety are proven in the specific local environment [257–259].

Post-market requirements are becoming more stringent, necessitating that developers provide regular RWP reports. This ensures that any performance degradation—whether due to changes in hospital scanner hardware or shifts in patient demographics—is identified and mitigated through re-training or recalibration under the established PCCP [257–259].

The ultimate objective of regulatory implementation is to achieve transparency in RWP. By consistently submitting RWP reports to regulatory authorities, developers can demonstrate that their algorithms remain robust within actual clinical workflows, independent of the initial validation sets. This process effectively completes the transition from “in-silico success” to “clinical trustworthiness” within a “closed-loop system.”

The deep intervention of AI technologies has triggered unavoidable socio-ethical issues, which have become institutional obstacles to clinical translation. Training high-precision models requires massive amounts of genomic, imaging, and clinical data from patients, thus creating severe risks with regards to privacy and the misuse of data. Balancing the use of big data for model optimization with the protection of patient privacy is an urgent legal challenge in the field of digital health. Many existing genomics and AI model training datasets were derived primarily from specific racial groups. Due to significant differences in genetic background between ethnic groups, applying these models directly to other populations may result in substantially reduced predictive efficacy, leading to “algorithmic discrimination” and exacerbating inequalities in the global allocation of medical resources. When human oversight is minimized or removed, issues of liability arise. For example, if an AI system makes an error, then questions will remain regarding who bears the responsibility in medical malpractice litigation and how this might impact on insurance coverage and liability scope. These are long-standing and unresolved issues that need to be addressed.

### Algorithmic bias and health equity challenges

As AI models move toward clinical deployment, addressing algorithmic bias is no longer a technical peripheral but a prerequisite for global health equity [224, 260–265].

Currently, a significant majority of the LC AI models described in this review (approximately 80-90%) have been developed and validated using datasets from Western cohorts (primarily from the US and Europe) or high-income East Asian countries [224, 260–265]. For instance, widely used benchmarks like the cancer genome atlas (TCGA-LUAD) and the national lung screening trial (NLST) consist predominantly of white and asian populations, with underrepresented groups such as black and hispanic populations often comprising less than 10% of the total sample [266]. This lack of diversity leads to “data poverty”, where models fail to generalize to the unique genetic landscapes and environmental exposures of diverse global populations [262, 267, 268]. Studies have shown that models trained exclusively on one ethnic group can experience a performance drop of 15–20% in AUC when tested on non-western or minority cohorts due to variations in baseline mutational burdens and distinct histological nuances [260, 265, 268–270].

For patients in low- and middle-income countries (LMICs), the deployment of “western-centric” AI carries high risks, primarily due to the following three reasons:(i) Genetic mismatch: models may misclassify tumors due to different prevalence rates of specific mutations. For example, EGFR mutation rates are significantly higher (up to 50%) in non-smoking asian populations compared to western cohorts (approx. 10–15%) [271–273]. (ii) Infrastructure and noise mismatch: Most models are optimized for high-resolution digital pathology or high-dose CT scans, which may not align with the lower-resource diagnostic equipment prevalent in LMICs, leading to unpredictable model behavior [265, 274–279]. (iii) Diagnostic Confounders: In many LMICs, LC screening is complicated by the high prevalence of co-infections such as tuberculosis. Models not exposed to these confounders during training may yield high false-positive rates [276, 280]. We advocate for fairness-aware machine learning and stratified reporting as per the latest transparent reporting of a multivariable prediction model for individual prognosis or diagnosis (TRIPOD) + AI guidelines to ensure that LC AI tools do not exacerbate existing healthcare inequities [281].

Federated learning (FL) offers a promising decentralized framework to mitigate these disparities [282–284]. Unlike traditional centralized training, FL allows a global model to be trained across multiple institutions without moving sensitive patient data [282–284]. Institutions in LMICs can contribute local data to a global “ensemble” model, ensuring that the AI learns from a diverse spectrum of ethnicities and clinical practices without the need for high-bandwidth data transfers. By keeping data localized, FL overcomes the legal and ethical barriers of cross-border data sharing, enabling a more inclusive representation of global health data while complying with local data sovereignty laws.

In summary, the implementation of multi-center and multi-ethnic validation across diverse racial groups and geographic regions has become a core requirement in the development and application of oncology AI to ensure the safety and equity of clinical deployment.

## Discussion and prospect

With the deep integration of AI technology and multimodal data, a new paradigm for the precision diagnosis and treatment of LC is likely to emerge. Transcending the limitations of single-omics data, AI efficiently integrates genomic driver mutations, transcriptomic microenvironmental features, radiomic macroscopic phenotypes, and dynamic signals from liquid biopsies to construct a panoramic tumor profile spanning from molecules to phenotypes. This transformation provides systematic tools for deciphering the high heterogeneity of LC, propelling the transition of clinical models from traditional empirical medicine to data-driven precision medicine, and may provide a core driving force for improving the prognosis of LC patients worldwide.

Currently, leading authoritative bodies such as the NCCN (National Comprehensive Cancer Network) and ESMO (European Society for Medical Oncology) have begun to focus deeply on AI and multi-omics technologies, issuing relevant framework guidelines or consensus statements [285–289]. However, the formal integration of these technologies into core clinical practice guidelines as “standard of care” remains in a transitional phase.

The spatiotemporal heterogeneity of LC stands as a pivotal factor contributing to therapeutic failure. Traditional single-source data modalities, such as sole reliance on CT imaging or solitary tissue biopsies, cannot capture the holistic landscape of a tumor. AI-driven multi-modal fusion strategies, such as the integration of radiomics with cfDNA fragment omics, achieve synergy between macroscopic phenotypes and microscopic molecular features. This cross-scale integration not only significantly reduces false-positive rates in LDCT screening and minimizes unnecessary invasive procedures but also offers the potential for precision subtyping and the prediction of immunotherapy response based on a digital biopsy for patients from whom tissue samples cannot be obtained. The convergence of AI and high-throughput sequencing technologies has elevated our understanding of LC biology to an unprecedented resolution. By integrating single-cell sequencing and ST data, AI models can systematically decode cellular interaction networks and spatial distributions within the TME; this is critical if we are to predict the efficacy of ICIs.

Multimodal AI integration offers a robust framework to bridge the gap between clinical phenotypes and underlying cell death mechanisms, such as ferroptosis, which plays a pivotal role in LC progression [290–293]. Recent studies have demonstrated that AI-driven signatures, such as the ferroptosis-related gene signature (FRGS) and lncRNA signature (FerRLSig), can accurately predict patient prognosis by decoding the complex interaction between ferroptosis-related markers and the TME [294–297]. By leveraging advanced computational frameworks such as DeepFerr—a deep learning model that utilizes neural networks to analyze the transcriptomic landscapes of key ferroptotic regulators—researchers can systematically identify molecular patterns of cell death evasion [298]. For instance, AI-driven analyses have successfully identified thioredoxin-interacting protein (TXNIP) as a central metabolic hub regulating ferroptosis in cancer stem cells, while subsequent studies have highlighted ATP-binding cassette subfamily C member 2 (ABCC2), ribonucleotide reductase M2 (RRM2) and aurora kinase A (AURKA) as critical suppressors that fortify tumors against ferroptotic induction [293, 298, 299]. These AI-derived insights effectively link macroscopic risk stratification with microscopic mechanisms of ferroptosis inhibition [293, 298, 299]. Such mechanistic elucidation paves the way for identifying novel synergistic targets capable of re-sensitizing recalcitrant LC cells to pro-ferroptotic therapies, thereby overcoming cross-resistance to chemotherapy and immunotherapy [293, 299–301]. Ultimately, this intelligent diagnostic ecosystem may provide a systematic tool for characterizing the “cold” or “hot” immune status of tumors based on their ferroptosis-associated oxidative stress profiles, guiding the development of precision therapeutic strategies to circumvent resistance to programmed cell death [293, 298, 299, 301].

The application boundaries of AI are accelerating from traditional in-hospital diagnosis and treatment to out-of-hospital rehabilitation and full lifecycle management. The core driver underlying this transition lies in the deep fusion of non-invasive multi-omics and dynamic real-time monitoring. Although single-cell sequencing and ST provide a refined histological atlas of the TME, their invasive nature limits clinical repeatability and longitudinal tracking. In contrast, the non-invasive or minimally invasive technologies provided by radiomics and liquid biopsy (encompassing genomics, metabolomics, and proteomics), provide a highly suitable alternative option for monitoring the evolution of disease due to their accessibility and repeatability. The unique advantage of liquid biopsy lies in its capability for full-cycle dynamic monitoring. By continuously analyzing body fluids such as blood and urine, clinicians can perform real-time assessments at every critical node, from disease screening and treatment response to recurrence and metastasis. Against this backdrop, an ideal eHealth paradigm is emerging. AI can not only decode micro-level liquid biopsy multi-omics data but also fuse this data with macro-level “passive sensing” data, such as vital sign fluctuations, captured by wearable devices. Through deep learning algorithms, these multi-modal data are transformed into clinically actionable digital biomarkers, thereby creating a closed-loop management system from precision in-hospital treatment to out-of-hospital rehabilitation monitoring, thus revolutionizing the dimensions of full-cycle disease management.

Although AI has demonstrated superior performance in a series of retrospective studies, the clinical application of this technology faces severe challenges, which can be summarized into the following three categories. First, technical bottlenecks; insufficient model interpretability (the “black box” problem) and limited generalization capabilities hinder trust and adoption by clinician. Second, data barriers; data privacy, algorithmic bias, and batch effects in sequencing data constitute institutional obstacles to multi-center collaboration., Third, the scarcity of clinical evidence; there is currently a lack of high-quality, prospective, RCTs to confirm the overall clinical utility of AI tools in real-world clinical pathways, specifically, their ability to tangibly improve diagnostic efficiency, reduce costs, and extend survival. Addressing these complexities and limitations, future research and translation should focus on three strategic directions, aiming to build a more inclusive, interpretable, and effective intelligent diagnostic and therapeutic ecosystem.

In order to further enhance the potential for the application of AI in the medical field, we need to focus on evolution from specialized models to foundation models (FMs). The future trend involves a shift from specialized models targeting single tasks (e.g., nodule detection) to general-purpose FMs that are trained by massive unlabeled data.

The landscape of AI in oncology is currently undergoing a transformative shift from task-specific models to general-purpose FMs [302–304]. Unlike traditional supervised learning that requires extensive expert labeling, FMs are pre-trained on massive-scale, unlabeled datasets using self-supervised learning (SSL) to capture universal biomedical representations [302–305]. Reflecting a profound paradigm shift since 2024, FMs such as UNI, CONCH, Prov-GigaPath, and CHIEF have redefined benchmarks in pathology AI [302–305]. These models transcend the limitations of traditional supervised learning by leveraging massive-scale, unannotated datasets to learn universal biological representations [302–305]. FMs may offer a transformative solution to the labeled-data scarcity problem in LC research [287, 306, 307]. By capturing inherent biological structures from massive amounts of unannotated data, FMs exhibit remarkable label efficiency, often requiring 50%–80% fewer labeled samples to achieve high diagnostic accuracy compared to task-specific models [287, 308–310]. Furthermore, their capacity for few-shot adaptation allows clinicians to identify rare LC subtypes or predict specific drug responses even when only a handful of labeled cases are available [287, 308–310]. Acting as a robust knowledge base, FMs facilitate the transfer of morphological and genomic patterns learned across diverse domains to specialized clinical questions, eliminating the need for exhaustive de novo training and significantly accelerating the translation of AI from research to precision oncology [287, 308–310].

Establishing a standardized multi-mode shared database and FLs mechanism is also crucial for the transformation of AI. The creation of large-scale, standardized repositories that integrate multi-omics with comprehensive follow-up data is a fundamental prerequisite for mitigating technical noise from sequencing platforms and ensuring efficient cross-modal alignment. To achieve this, unified standards for data collection and preprocessing must be formulated to normalize inherent heterogeneity. Simultaneously, FL should be promoted as a critical technical framework for robust data governance, addressing the primary obstacles of data privacy and ethical restrictions. Unlike traditional centralized learning, FL empowers multiple medical institutions to collaboratively train AI models by exchanging locally computed model updates such as gradients or weights rather than sensitive raw patient data. By facilitating multi-institutional collaboration while strictly safeguarding patient confidentiality, this decentralized approach effectively dismantles “data silos,” thereby enhancing the global applicability, robustness, and healthcare equity of AI-driven clinical systems [311–313].

The transition from fragmented “data silos” to a “standardized multi-modal shared database” is a critical technical hurdle. Achieving this requires a multi-layered harmonization strategy across technical, syntactic, and semantic domains. Central to this approach is the rigorous adherence to international interoperability protocols, such as digital imaging and communications (DICOM) for imaging, fast healthcare interoperability resources (FHIR) for clinical records, and the FAIR principles (Findable, Accessible, Interoperable, Reusable) to ensure robust data stewardship [314–320]. However, implementation must navigate complex legal hurdles like general data protection regulation (GDPR) and health insurance portability and accountability act (HIPAA), which necessitate privacy-preserving technologies such as FL to maintain data sovereignty while enabling cross-border collaboration. By learning from successful initiatives like TCGA and EuCanImage —and avoiding the pitfalls of unannotated “Data Swamps”—we propose a Four-Stage Harmonization Framework [321–324]: (i) Ingestion Layer: Automated extraction using ETL (Extract, Transform, Load) pipelines to convert raw data into Common Data Models (CDM), such as OMOP. (ii) Semantic Layer: Mapping disparate terminologies to standardized ontologies (e.g., SNOMED CT for clinical findings, LOINC for labs). (iii) Fusion-Ready Layer: Temporal alignment of longitudinal data (aligning CT scans with biopsy dates and treatment cycles). (iv) Governance Layer: Implementation of blockchain-based audit trails or smart contracts to manage data access and maintain the “chain of custody.”

Last but not least, we must strengthen prospective clinical validation and XAI. To drive AI technology across the chasm from “technical validation” to “clinical benefit”, the focus of future research must shift from retrospective validation relying solely on historical data (in silico) to designing rigorous prospective RCTs. This paradigm shift is essential to objectively evaluate the actual efficacy of AI tools within real-world clinical workflows. Concurrently, to dismantle the “black box” barrier and facilitate deep human-machine collaboration, emphasis should be placed on leveraging emerging XAI techniques such as saliency maps for pixel-level importance and attention visualizations for highlighting critical data relationships [204–207]. By integrating these techniques with prior biological knowledge to intuitively visualize key features—including metabolic nodes and spatial cell interactions—XAI can significantly enhance model transparency and logical credibility. Such interpretability is vital for accelerating the safe and ethically sound implementation of AI in clinical decision-making.

The deep fusion of AI and multi-modal data is reshaping every aspect of clinical LC management, constructing a panoramic tumor cognitive profile from molecules to phenotypes. This technological revolution not only optimizes early screening, molecular subtyping, and immunotherapy strategies but also participates in the dynamic management of postoperative rehabilitation. However, the leap of AI from research to clinical practice is critical, encompassing the entire process from data collection and algorithm deployment to ethical regulation. In the future, solving data standardization and sharing issues, breaking “data silos” to eliminate algorithmic bias, while simultaneously developing interpretable AI models and conducting rigorous multi-center prospective RCTs, will be the inevitable path to ultimately achieving efficient, equitable, and precise diagnosis and treatment for LC.

## Supplementary information

List of abbreviations
