# Supplementary material for: A new era of precision diagnosis and treatment for lung cancer: artificial intelligence-driven multimodal data integration and clinical applications
Source: Cell Death Dis. 2026 Apr 22;17(1):534. doi: 10.1038/s41419-026-08769-z (PMC13237044; doi:10.1038/s41419-026-08769-z)
Supplement: Supplementary file 1 — List of abbreviations [file 41419_2026_8769_MOESM1_ESM.docx]

**List of abbreviations**

| **Abbreviation** | **Full name** |
| --- | --- |
| AI | Artificial intelligence |
| LC | Lung cancer |
| TME | Tumor microenvironmen |
| CNNs | Convolutional Neural Networks |
| GNNs | Graph Neural Networks |
| DL | Deep Learning |
| ML | Machine learning |
| NSCLC | Non-Small Cell Lung cancer |
| LUAD | Lung Adenocarcinoma |
| SCLC | Small Cell Lung cancer |
| LDCT | Low-dose computed tomography |
| CAD | computer-aided diagnosis |
| GWAS | Genome-wide association studies |
| PRS | Polygenic risk scores |
| NGS | Next-generation sequencing |
| cfDNA | Cell-free DNA |
| SVM | Support Vector Machines |
| 5hmC | 5-hydroxymethylcytosine |
| AUC | Area under the curve |
| EVs | Extracellular vesicles |
| ELISA | Enzyme-Linked Immunosorbent Assay |
| WSIs | whole-slide images |
| OS | overall survival |
| ICB | Immune checkpoint blockade |
| PFS | Progression-free survival |
| scRNA-seq | Single-cell RNA sequencing |
| GATs | Graph Attention Networks Graph Attention Networks |
| ST | Spatial transcriptomics |
| ICIs | Immune checkpoint inhibitors |
| ANN | Artificial Neural Network |
| TIME | Tumor Immune Microenvironment |
| mIHC | multiplex Immunohistochemistry |
| eHealth | Digital health |
| EMR | Electronic Medical Records |
| RCTs | Randomized controlled trials |
